# Expression of zebrafish *pax6b *in pancreas is regulated by two enhancers containing highly conserved *cis*-elements bound by PDX1, PBX and PREP factors

**DOI:** 10.1186/1471-213X-8-53

**Published:** 2008-05-16

**Authors:** François M Delporte, Vincent Pasque, Nathalie Devos, Isabelle Manfroid, Marianne L Voz, Patrick Motte, Frédéric Biemar, Joseph A Martial, Bernard Peers

**Affiliations:** 1 Unit of Molecular Biology and Genetic Engineering, University of Liège, Giga-R, B34, Avenue de l'hôpital, 1, B-4000 Liège, Belgium; 2 Laboratory of Plant Cell and Molecular Biology, University of Liège, Department of Life Sciences, Institute of Botany, B-4000 Liège, Belgium; 3 Department of Biology, Temple University, Philadelphia, PA 19122, USA; 4 Wellcome/Cancer Research UK Gurdon Institute, University of Cambridge Tennis Court Road, Cambridge CB2 1QN, U.K

## Abstract

**Background:**

PAX6 is a transcription factor playing a crucial role in the development of the eye and in the differentiation of the pancreatic endocrine cells as well as of enteroendocrine cells. Studies on the mouse *Pax6 *gene have shown that sequences upstream from the P0 promoter are required for expression in the lens and the pancreas; but there remain discrepancies regarding the precise location of the pancreatic regulatory elements.

**Results:**

Due to genome duplication in the evolution of ray-finned fishes, zebrafish has two *pax6 *genes, *pax6a *and *pax6b*. While both zebrafish *pax6 *genes are expressed in the developing eye and nervous system, only *pax6b *is expressed in the endocrine cells of the pancreas. To investigate the cause of this differential expression, we used a combination of *in silico*, *in vivo *and *in vitro *approaches. We show that the *pax6b *P0 promoter targets expression to endocrine pancreatic cells and also to enteroendocrine cells, retinal neurons and the telencephalon of transgenic zebrafish. Deletion analyses indicate that strong pancreatic expression of the *pax6b *gene relies on the combined action of two conserved regulatory enhancers, called regions A and C. By means of gel shift assays, we detected binding of the homeoproteins PDX1, PBX and PREP to several *cis*-elements of these regions. In constrast, regions A and C of the zebrafish *pax6a *gene are not active in the pancreas, this difference being attributable to sequence divergences within two *cis*-elements binding the pancreatic homeoprotein PDX1.

**Conclusion:**

Our data indicate a conserved role of enhancers A and C in the pancreatic expression of *pax6b *and emphasize the importance of the homeoproteins PBX and PREP cooperating with PDX1, in activating *pax6b *expression in endocrine pancreatic cells. This study also provides a striking example of how adaptative evolution of gene regulatory sequences upon gene duplication progressively leads to subfunctionalization of the paralogous gene pair.

## Background

The pancreas has two major functions fulfilled by distinct tissues: i) production of digestive enzymes by the exocrine cells and ii) release of various hormones by distinct endocrine cell types (i.e. secretion of glucagon, insulin, somatostatin, pancreatic polypeptide and ghrelin by α, β, δ, PP and ε cells, respectively). These mature pancreatic endocrine and exocrine cells derive from a pool of endodermal progenitor cells located in the embryonic gut. Differentiation of these cells is controlled by a regulatory cascade involving a battery of pancreatic transcription factors (reviewed in [1,2]). The HOX-like homeoprotein PDX1 is expressed in pancreatic progenitor cells and plays a crucial role in pancreas development. Its absence results in an early arrest of pancreatic bud growth and blocks both exocrine and endocrine cell differentiation [3-5]. PDX1 regulates the expression of downstream target genes by acting in concert with regulatory proteins, including other homeodomain proteins such as PBX and MEIS/PREP [3-9]. Subsequent commitment of PDX1^+ ^pancreatic progenitors to the endocrine lineage is controlled by a set of other transcription factors such as NGN3, NEUROD, ISL1 and INSM1/IA1 [10-13]. Specification of the various endocrine cell subtypes involves the action of downstream regulators such as the homeodomain-containing proteins PAX6, PAX4, ARX, NKX2.2 and NKX6.1 [14-18]. In mouse, *Pax6 *is expressed in all pancreatic endocrine cells and its disruption leads to a reduction of α, β and δ cells and an increase of ε cells [17,19,20]. *Pax6 *plays crucial functions in the development of several organs/tissues besides the endocrine pancreas, such as the eyes, olfactory system, brain, spinal cord, enteroendocrine cells and the pituitary (reviewed in [21-24]).

Expression of the *Pax6 *gene is controlled by a highly complex system of regulatory elements comprising at least three distinct promoters (P0, P1 and Pα) and many *cis*-acting elements or enhancers, located along the gene [25-27]. Long-range control elements have been identified in the human *PAX6 *locus located more than 150 kb downstream from the transcription unit [28]. Sequence comparisons of different vertebrate *Pax6 *genes have revealed that most of these *cis*-acting elements are evolutionarily conserved. Reports published so far indicate that expression in a specific tissue can be driven by several distinct enhancers. Experiments focusing on the zebrafish, quail, mouse, and human *Pax6 *genes have highlighted several distinct enhancers targeting expression to the neuroretina. *In vitro *experiments with the quail *Pax6 *gene have revealed a region located 7.5 kb downstream from the quail P0 promoter, acting as an enhancer in neural retina cells [29]. Several *in vivo *transgenic studies have led to identification of at least five other retinal enhancers, located respectively at 2 kb upstream from the murine P0 promoter [30,31], just upstream from the promoter Pα [30,32-34], within intron 7 [35], and even 70 kb and 100 kb downstream from the *Pax6 *gene [35,36].

Two enhancers have been identified as participating in the control of *Pax6 *expression in the lens. The first, driving expression in the lens placode and corneal ectoderm, lies about 3.5 kb upstream from the P0 murine promoter [31,37-40] and is recognized by a multi-protein complex composed of the homeoproteins MEIS1 and MEIS2 [40]. This enhancer is also bound by a complex containing the SRY-like HMG box SOX2 transcription factor and the OCT-1 factor, which are essential for *Pax6 *expression in the lens placode and its derivatives [41]. The second lens enhancer consists of the EI/SIMO elements located 80 kb downstream the last *Pax6 *exon [36].

Regulatory regions involved in pancreatic expression have also been identified within the *Pax6 *locus. Two different groups have shown that in the mouse, the pancreatic regulatory elements are located upstream from the P0 promoter [31,32,42]. These groups disagree, however, as to the precise location of these elements: Kammandel and coworkers observed disrupted pancreatic expression of the *LacZ *reporter gene after deletion of sequences located 4 kb upstream from exon 0, indicating the presence of an essential pancreatic element in this region [32]. In contrast, Zhang and co-workers found the first 2.3 kb of the *Pax6 *P0 promoter to be sufficient for expression in the pancreas and observed that a further 400 bp 5' deletion of the promoter entirely abolished reporter expression [31]. The reason for this discrepancy is still unknown.

In zebrafish, partial duplication of the genome during teleost evolution has led to two *pax6 *paralogs named *pax6a *and *pax6b *[26,43]. Both genes are expressed in overlapping areas during development. They are activated in the anterior neural plate at the end of gastrulation; however, their expression pattern diverges slightly at later stages [44,45]. Although the embryonic expression patterns of the two zebrafish *pax6 *genes have been well-characterized in ocular structures and the CNS, few data are available on their pancreatic expression. We have previously reported expression of *pax6b *in the pancreas at early stages (15–24 hpf) [46], but whether *pax6a *gene is activated in the pancreas at later developmental stages is still unclear.

In the present study, we have compared in detail the expression of both zebrafish homologs. We show that only *pax6b *is expressed in the pancreas during embryogenesis and we highlight *cis*- and *trans*-regulatory elements responsible for this differential pancreatic expression.

## Results

### Zebrafish *pax6b *is expressed in endocrine pancreatic cells in contrast to the zebrafish *pax6a *gene

**Figure 1:**
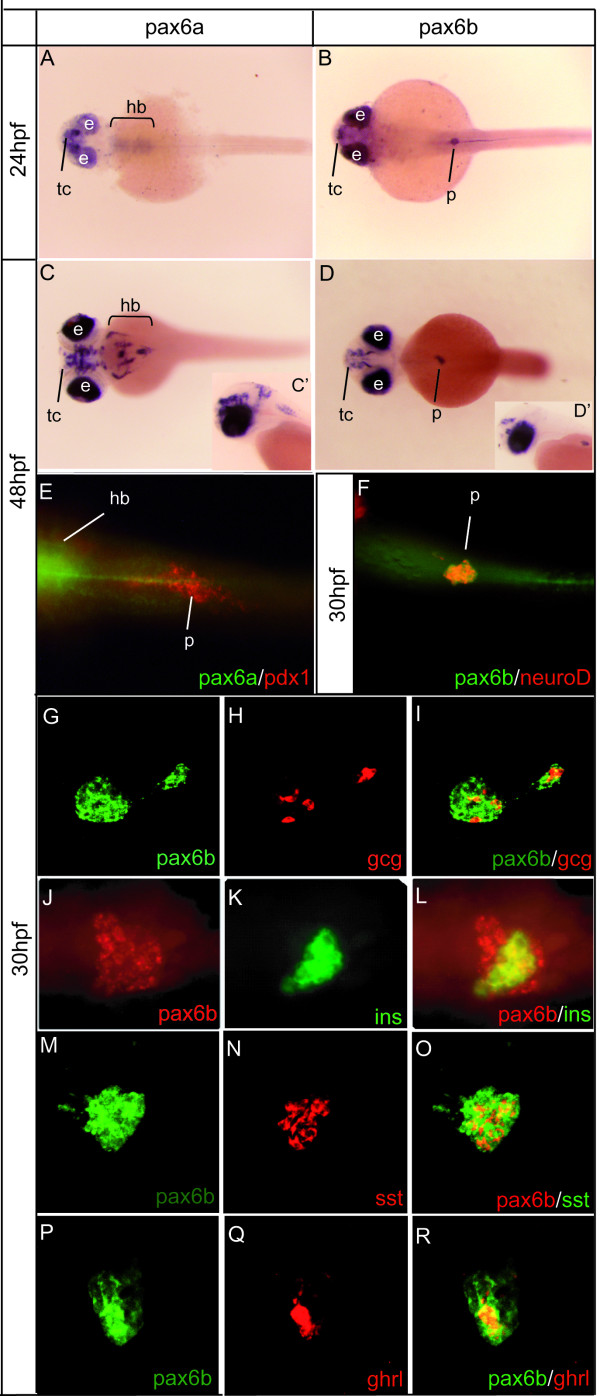
**Zebrafish *pax6b *is expressed in endocrine pancreatic cells in contrast to the zebrafish *pax6a *gene**. **(A, B) **At 24 hours of development, both *pax6a *and *pax6b *mRNAs are detected by *in situ *hybridization in the eye and in the telencephalon but only *pax6b *is expressed in the pancreas. **(C-D') **At 48 hours post fertilization (hpf), *pax6a *and *pax6b *are expressed in overlapping areas, such as the ocular stuctures (retina and lens), the ventral telencephalon and the diencephalon. *pax6a *is detected more strongly in the hindbrain whereas only *pax6b *is expressed in the pancreatic endocrine islet. **(E) ***pax6b *colocalizes with neurod in the differentiating endocrine cells by 30 hpf. (F) No pancreatic expression of *pax6a *is detected at 48 hpf, as demonstrated by double WISH with *pax6a *and *pdx1*. **(G-R) **Double fluorescent *in situ *hybridizations on 30 hpf zebrafish embryos show *pax6b *colocalization with the four major hormones glucagon, insulin, somatostatin and ghrelin. Abbreviations: **e**, eye; **gcg**, glucagon; **ghrl**, ghrelin; **hb**, hindbrain; **ins**, insulin; **p**, pancreas; **sst**, somatostatin; **tc**, telencephalon. Embryos in figures A-R are presented in a ventral view, anterior to the left. Embryos in C'-D' are presented in a lateral view, anterior to the left.

We first compared, by whole mount *in situ *hybridization (WISH), the temporal and the spatial expression patterns of the *pax6a *and *pax6b *genes between the 10-somite stage and 5 days of zebrafish development (Fig. 1). At 10-somite stage, we found both genes to be expressed in the eye field, the presumptive diencephalon and hindbrain and the spinal cord (data not shown) [24,43]. By 24 onward, clear differences in expression were noted between the two genes at the level of the hindbrain and pancreas, *pax6a *being expressed more strongly than *pax6b *in the hindbrain while only *pax6b *was detected in the pancreatic anlagen at all stages examined (Fig. 1A–D'). To further characterize the expression of *pax6a *and *pax6b*, double-fluorescent WISH was carried out. At 30 hpf, *pax6b *colocalizes with *neuroD *expressed in all pancreatic endocrine cells (Fig. 1F). As mentioned above, *pax6a *was never detected in the pancreas as revealled by the absence of colocalization with the pancreatic marker *pdx1 *(Fig. 1E). At the pancreatic level, the results clearly show that *pax6b *is expressed in the α, β, δ and ε endocrine cell types (Fig. 1G–R).

### Sequence comparison of vertebrate *pax6 *genes reveals three conserved regions upstream from the P0 promoter

**Figure 2:**
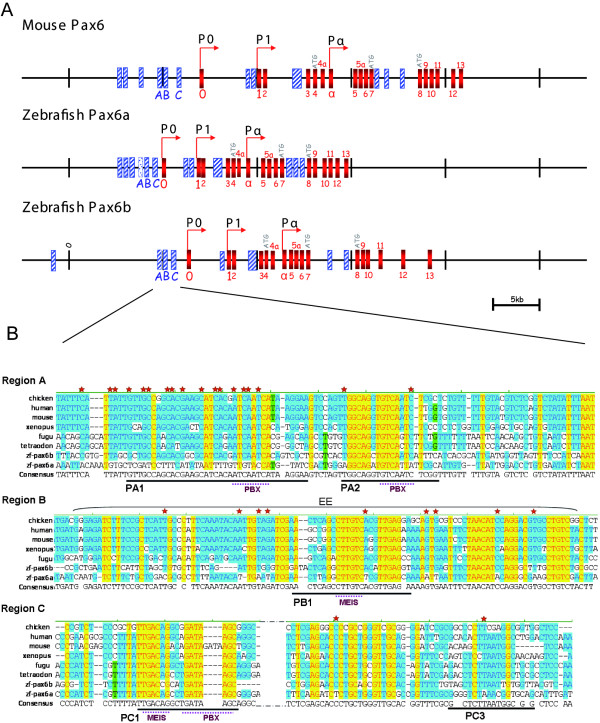
**Sequence comparison of vertebrate *Pax6 *genes reveals three conserved regions upstream from the P0 promoter**. **(A) **Genomic organisation of the mouse and zebrafish *Pax6 *genes composed of 17 exons (red boxes) and three promoters P0, P1 and Pα (arrows). Comparison of the three gene sequences revealed evolutionary conserved non-coding regions (blue boxes), notably three regions A, B and C located upstream the P0 promoter. Scale bar: 5 kb. **(B) **Multiple sequence alignment of the three conserved regions A, B and C from the human, mouse, chick, xenopus, fugu, tetraodon (except the B region, not found), and both zebrafish *pax6 *genes. The colour within the sequence alignment indicates the degree of similarity: yellow indicates complete identity between all species; blue indicates identity for at least 50% of the different sequences. Red stars represent nucleotide positions conserved in all vertebrates (including *pax6b*) but divergent in *pax6a*. Consensus sites for the binding of PBX1 and/or MEIS are underlined. PA1, PA2, PC1 and PC3 show the location of the conserved DNA elements used in EMSAs. EE corresponds to the lens/ectodermal enhancer described by Zhang et al, 2002.

To identify the *cis*-regulatory elements responsible for *pax6b *expression in the pancreas, we first aligned the sequences of the two zebrafish *pax6 *genes with the mouse *Pax6 *gene. Both zebrafish *pax6 *genes have a structure similar to that reported for the mouse and human *PAX6 *genes (Fig. 2A) [24,45,47,48]. A comparison of the zebrafish genomic sequence with cDNA and EST sequences revealed the presence of at least 17 exons. The location of the 5' ends of the *pax6b *transcripts confirms the presence of three promoters, P0, P1 and Pα as described for the mouse and human genes. A comparison of the zebrafish *pax6a *and *pax6b *genes with the mouse *Pax6 *gene revealed that most exons are conserved (red boxes in Fig. 2A). Conserved regions were also found in non transcribed sequences (blue boxes in Fig. 2A), most of which are located within previously described regulatory regions. Interestingly, some of these regulatory sequences are more conserved in one of the two zebrafish *pax6 *genes and thus might be responsible for the slightly different expression patterns. For example, one conserved element in the P1 promoter and one element in intron 7 are well conserved in *pax6a *but not in *pax6b*. Conversely, one element upstream from the P0 promoter is well conserved in the *pax6b *but not in *pax6a *(see box A in Fig. 2A).

To identify the sequence motifs of the P0 promoter conserved among all known vertebrate *Pax6 *genes, we performed multiple alignments of the sequences encompassing conserved regions A, B, C (see Fig. 2B). In the figure, the nucleotide positions that are identical in all species are shown in yellow, while those conserved in all vertebrate *Pax6 *genes except zebrafish *pax6a *are highlighted by red stars. These alignments clearly show that region A is less conserved than regions B and C in the zebrafish *pax6a *gene, especially at the level of one particular motif, named PA1. Region A actually corresponds to the pancreatic element reported for the mouse *Pax6 *gene by Kammandel and coworkers [32]. Region B overlaps with the lens placode enhancer [32,39,40] and region C maps to the pancreatic element reported by the group of Maas [31,42]. As expression in the pancreas is observed for all vertebrate *Pax6 *genes except zebrafish *pax6a*, sequence divergence within region A might be the cause of this differential expression. This would be in agreement with Kammandel's results locating a pancreatic regulatory element in region A.

### The upstream regulatory region of the zebrafish P0 *pax6b *promoter targets expression to the endocrine pancreas

**Figure 3:**
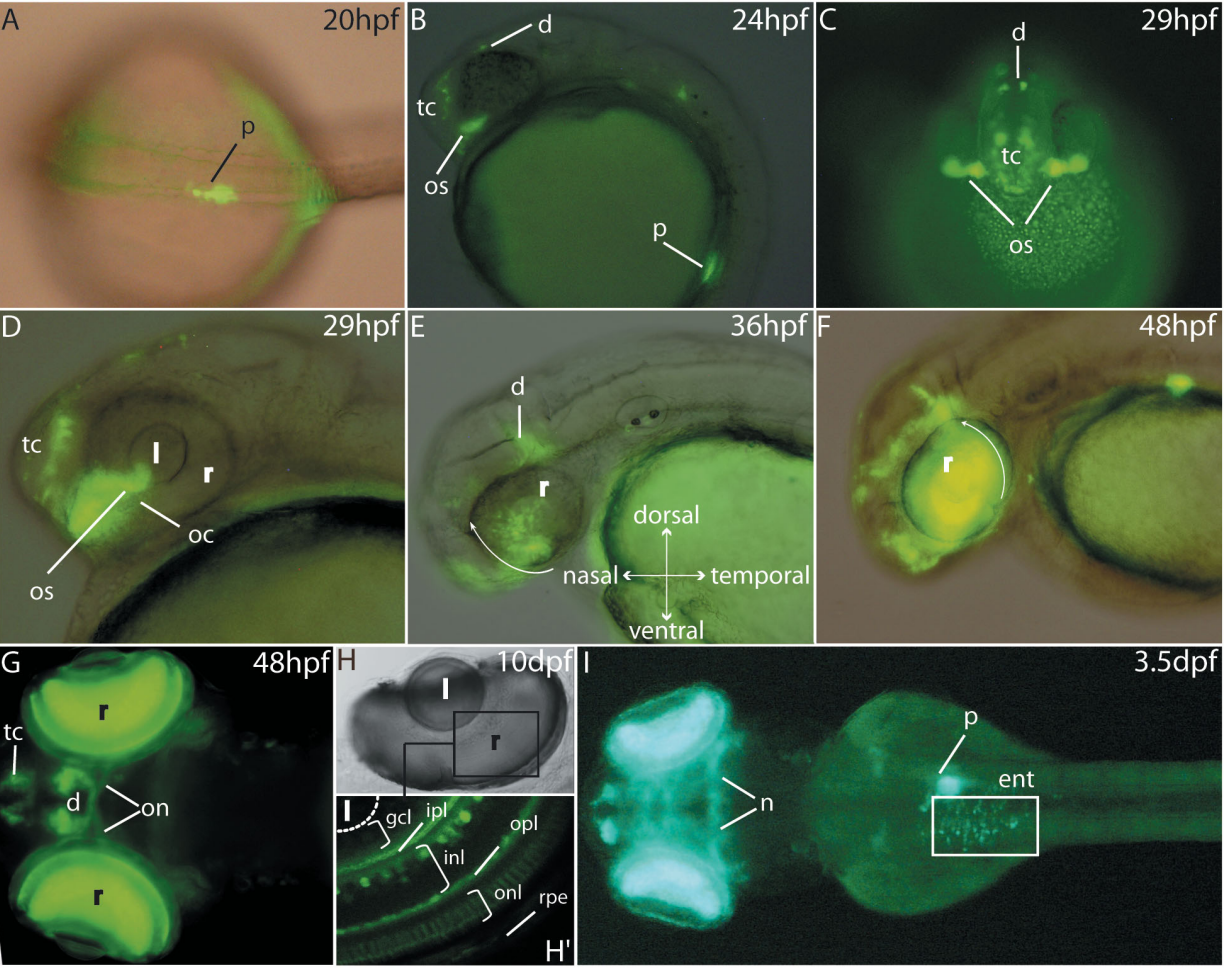
**GFP expression pattern in the stable P0-*pax6b*:*gfp *transgenic zebrafish**. **(A) **Dorsal view of a 20 hpf transgenic embryo showing GFP expression in the developing pancreas. **(B, C) **Lateral and frontal views at 24 and 29 hpf showing GFP expression in the ventral telencephalon, the dorsal diencephalon and the optic stalks. **(D, E, F) **Lateral views of an eye of 29 hpf, 36 hpf and 48 hpf transgenic embryos. GFP expression starts at about 29 hpf in a small cluster of cells in the ventronasal retina, near the optic stalks, and subsequently spreads to the entire retina (white arrows). **(G) **After 2 days of development, expression is observed in the neuroretina, the optic nerves as well as the ventral telencephalon and some cells of the diencephalon. **(H, H') **At 10 dpf, expression is clearly detected in several layers of the retina, mainly the inner plexiform layer, the inner nuclear layer, the outer plexiform layer and the outer nuclear layer. **(I) **Finally, from 3.5 dpf, the transgenes are expressed in the enteroendocrine cells of the intestine and in some neurones of the mesencephalon. Abbreviations: **d**, diencephalon; **e**, eye; **ent**, enteroendocrine cells; **gcl**, ganglion cell layer; **hb**, hindbrain; **in**l, inner nuclear layer, **ip**l, inner plexiform layer; **l**, lens; **mb**, midbrain; **nt**, neural tube; **oc**, optic choroid; **on**, optic nerves; **onl**, outer nuclear layer; **opl**, outer plexiform layer; **os**, optic stalks; **p**, pancreas; **pcl**, photoreceptor cell layer; **r**, retina; **rpe**, retinal pigmented epithelium; **tc**, telencephalon. A, G and I are dorsal views (anterior to the left); B, D, E and F are lateral views (anterior to the left); H, H' are cross sections in the eye of a 10 dpf embryo.

To determine whether the P0 promoter of *pax6b *can target expression to the pancreas, we fused 3.8 kb of the *pax6b *P0 promoter, including the conserved regions A, B and C, upstream from the *gfp *or *dsred *coding region. These two reporter constructs were injected into fertilized zebrafish eggs and the resulting embryos were analysed for GFP/DSRED expression 24, 48 and 75 hours after injection. Transgene expression was detected in cells of the retina, brain and pancreas (data not shown). To establish stable transgenic lines, injected embryos displaying high GFP or DSRED expression were raised to adulthood and then tested for germline transmission of the *pax6b *transgene. We identified one *pax6b:gfp *founder and five *pax6b:dsred *founders. Embryos generated from all these founders displayed very similar expression patterns. The only differences observed between lines concerned the level of transgene expression. Expression of the *pax6b *transgenes is first detected in the pancreatic anlagen around 14-somite stage, and is clearly visible by 20 hpf in all transgenic lines (Fig. 3A). Between 24 and 29 hours post fertilization, GFP/DSRED also appears in the ventral telencephalon, the optic stalks and the dorsal diencephalon (Fig. 3B, 3C).

About 29 hpf, first signs of GFP/DSRED expression also appear in a small cluster of cells in the ventronasal part of retina, near the optic stalks and the optic choroid (Fig. 3D). This expression gradually expands and spreads to the entire retina, from the ventral to the nasal retina first and subsequently to the temporal and dorsal regions (arrows in Fig. 3E, 3F). This expression pattern is reminiscent of the wave of neurogenesis occurring in the retina [49] and coincides spatiotemporally with the differentiation wave of retinal ganglion cells (RGC). On day 2 of development, transgene expression is also detected in the axons of the RGC within the optic nerves, revealing the optic chiasma (Fig. 3G). In 10 dpf embryos, GFP/DSRED expression is detected in several layers of the neuroretina corresponding mainly to the inner plexiform layer, subset of cells in the inner nuclear layer (most likely the amacrine cells), the outer plexiform layer and to the outer nuclear layer (Fig. 3H, H'). From 3.5 dpf onwards, *pax6b *transgenes are also detected in scattered cells of the gut corresponding to the enteroendocrine cells and in some neurons of the mesencephalon (Fig. 3I). Expression of the transgenes gradually disappears in pancreas by 9 dpf, and in the enteroendocrine cells by about 14 dpf, while being maintained in the other tissues, including neurons of the brain and some layers of the retina (data not shown).

### Pancreatic expression of *pax6b *relies on the two highly conserved regions A and C

**Figure 4:**
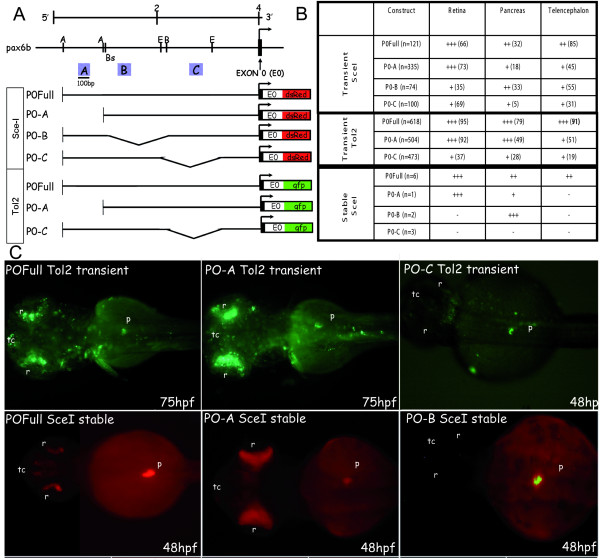
**GFP/DSRED expression in transient and stable zebrafish transgenic embryos obtained with deletion constructs of *pax6b *P0 promoter**. **(A) **Restriction map of the zebrafish *pax6b *genomic locus. The arrow indicates the transcriptional start site at exon 0 (E0, marked in black). Promoter deletion constructs were made by enzymatic digestion at the sites indicated. A, B and C conserved regions of approximately 100-bp long are indicated by blue boxes. **(B) **These DNA constructs were injected into zebrafish embryos and DSRED (*Sce*-I transgenesis) or GFP (Tol2 transgenesis) transient expression was analysed at 24, 48 and 75 hours of development. The percentage of embryos expressing DSRED/GFP in the retina, the pancreas and the telencephalon versus the total number of embryos expressing DSRED/GFP (n) is indicated in brackets; the number of DSRED/GFP-expressing cells in each tissue (from +++ to +) is shown in the table. For the stable transgenic lines, n is the number of different transgenic founders obtained, and the level of DSRED expression is indicated (from +++ to -). **(C) **Transient GFP expression in 75 and 48 hpf embryos injected with the full PO (FullP0), the A-deleted (P0-A) and the C-deleted (PO-C) constructs using Tol2-mediated transgenesis. DSRED expression in stable transgenic embryos at 48 hpf harboring the full P0 construct, the A-deleted construct or the B-deleted construct after *Sce*-I transgenesis. Abbreviations: **A**, *Acc*I; **B**, *Bcl*I; **Bs**, *Bst11071*I; **E**, *EcoN*I; **p**, pancreas; **r**, retina; **tc**, telencephalon. All the embryos are presented in dorsal views with anterior on the left.

To pinpoint regulatory regions essential to expression in these various tissues, we generated deletions of the conserved regions A, B and C and tested the corresponding constructs in both transient and stable transgenic zebrafish (Fig. 4A). For the transient expression approach, we used two different transgenesis methods: the *Sce*-I meganuclease method [50] and transposon Tol2-mediated transgenesis [51]. The embryos were analysed 24 and 48 hours after transgene injection, and the percentage of injected embryos displaying GFP/DSRED expression in the retina, telencephalon, and pancreas was determined (Fig. 4B). With the *Sce*-I method, about half of the embryos injected with the full length construct showed DSRED expression, 66% of which displayed it in the retina, 32% in the pancreas and 85% in the telencephalon. The observed transient and mosaic expression is in agreement with the expression detected in the stable lines. Deletion of enhancer A led to a decrease of the percentage of embryos expressing DSRED in the endocrine pancreas, from 32 to 18%, and to reduction of the number of DSRED expressing cells in this tissue (from ++ to +). When region B was deleted, the proportion of embryos expressing the reporter protein in the retina and telencephalon was significantly reduced, while expression in the pancreas remained unchanged (Fig. 4B). Deletion of region C led to a strong decrease in DSRED positive embryos, and the percentage of embryos expressing DSRED in the pancreatic tissue was reduced from 32 to 5%.

When the Tol2-transposon method was used, the efficiency of the transgenesis was drastically increased, and about 95% of the embryos injected with the full length P0 construct transiently expressed GFP in the retina; 79% expressed it in the pancreas, and 91% expressed it in the telencephalon (Fig. 4B, 4C), showing that Tol2-mediated transient expression reproduces fairly well the expression pattern detected in the stable transgenic line. Deletion of element A led to a decrease in the number of embryos expressing GFP in the pancreatic islet (from 79 to 49%), suggesting that this element is necessary for high pancreatic expression. When we removed enhancer C, the overall transgene expression was greatly diminished and expression in the pancreas was also reduced to only 28% of injected embryos (Fig. 4B, 4C).

For each deletion construct used for *Sce*-I transgenesis, the injected embryos were raised to adulthood and tested for germline transmission (Fig. 4B, 4C). One transgenic line was obtained for the A-box deletion construct. While DSRED expression was still clearly observed at the level of the retina and enteroendocrine cells in this transgenic line, much weaker expression was observed in the pancreas as compared to the transgenic lines harboring the full-length P0 promoter construct (Fig. 4C). Two stable transgenic lines were obtained with the B-box deletion construct. These two lines displayed very strong DSRED expression in the pancreas but no detectable expression in the retina, the enteroendocrine cells or the telencephalon (Fig. 4B, 4C). Finally, three stable lines harboring the C-box deletion construct were identified by PCR but none of them displayed detectable DSRED expression (data not shown).

Taken together, these transient and stable expression data indicate that region A is required for expression in the telencephalon and for maintaining a high expression level in the pancreas, while element B is crucial to retinal expression. Enhancer C is crucial to the overall activity of the P0 promoter, since transgene expression was prevented in stable transgenic lines or strongly reduced in transient expression assays, when this region was missing. Moreover, the results of the *Sce*-I transient expression assays suggest that region C may play a role in pancreatic expression.

**Figure 5:**
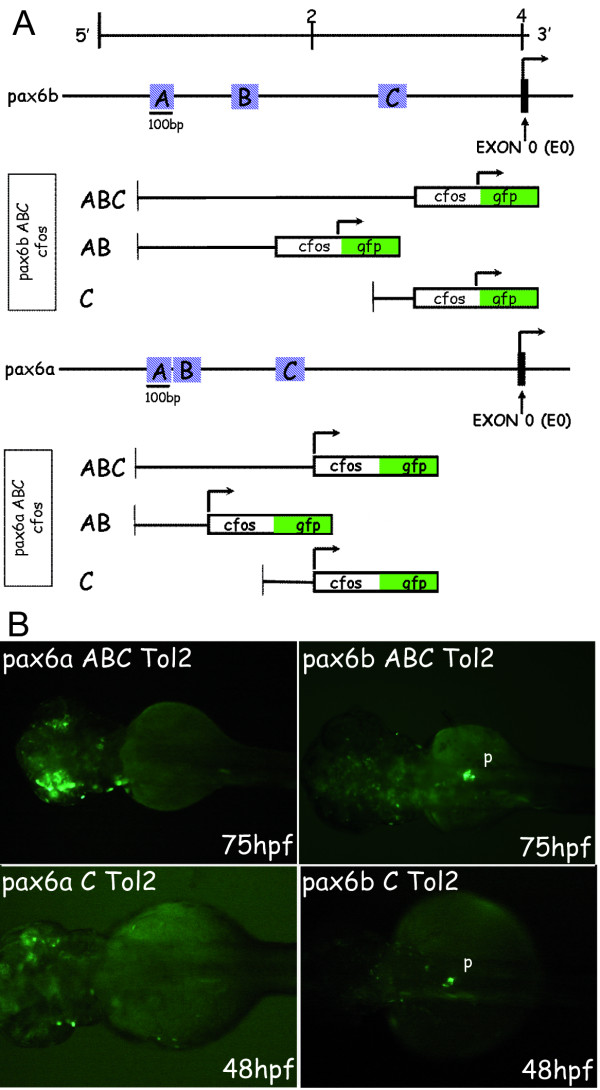
**GFP expression in transient zebrafish transgenic embryos obtained with constructs carrying *pax6b *and *pax6a *AB and/or C regions fused to the *cfos *heterologous promoter**. **(A) **Restriction map of the zebrafish *pax6b *and *pax6a *genomic locus. The arrow indicates the transcriptional start site at the exon 0 (E0, marked in black). For the constructs, the different regions to test were fused to the *cfos *heterologous promoter. The arrow indicates the transcriptional start site at the *cfos*. Fusion constructs (AB-*cfos *and C-*cfos*) were made by PCR using specific primers (see materials and methods for sequences). These DNA constructs were injected into zebrafish embryos using Tol2 mediated transgenesis and GFP transient expression was analysed at 24, 48 and 75 hours of development. **(B) **Transient GFP expression in 75 and 48 hpf embryos injected with the full ABC-*cfos *and the C-*cfos *constructs. All the embryos are presented in dorsal views with anterior on the left. Abbreviations: **p**, pancreas.

To further determine the roles of these regions, we examined whether they are sufficient for driving pancreatic expression. For this we produced constructs containing regions A, B and C of the zebrafish *pax6 *genes cloned in front of a minimal *cfos *promoter fused to the *gfp *sequence (*cfos*-*gfp*) and flanked by Tol2 inverted repeats (Fig. 5A). These constructs were injected in zebrafish eggs with Tol2 transposase and the embryos were tested for GFP expression at 48 and 75 hpf (Fig. 5B). We found that the region ABC of *pax6b *can drive GFP expression in the pancreas but that the homologous region of *pax6a *cannot (Fig. 5B). This demonstrates that the differential pancreatic expression of the two zebrafish *pax6 *genes is due to sequence differences within the conserved ABC regions. It also should be noted that mosaic expression of *pax6b*-ABC-*cfos *transgene was detected within the retina (data not shown). In experiments where either the C region or the AB regions were inserted alone in front of the heterologous *cfos *promoter, we observed that the C region of *pax6b *was sufficient to drive some GFP expression in the pancreas, whereas that of *pax6a *was not (Fig. 5B). Yet the level of pancreatic expression observed was much lower with *pax6b *region C alone than with the *pax6b *ABC construct. No pancreatic expression was observed with the construct carrying regions A and B of either *pax6a *or *pax6b *(data not shown). Altogether, these data indicate that high pancreatic expression relies on the combined action of regions A and C.

### The PA1 and PA2 elements of *pax6b *are recognized respectively by a cellular complex containing the PDX1-PBX-PREP trimer or the PBX-PREP dimer

**Figure 6:**
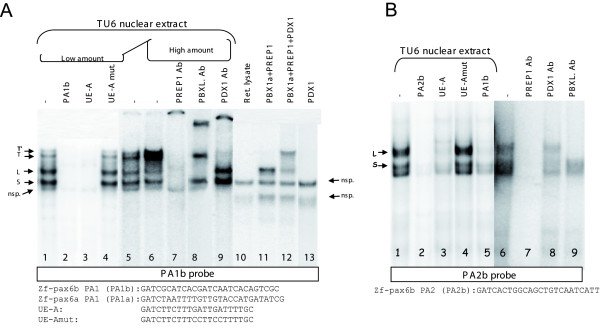
**PBX and PREP homeoproteins bind to the P0-*pax6b *PA2 element and with PDX1 to the P0-*pax6b *PA1 element**. **(A) **EMSAs were performed on PA1 probe of *pax6b *(PA1b) using nuclear extract of pancreatic TU6 cells. 10 ng of unlabeled oligonucleotides PA1b, UE-A or mutated UE-A (sequences depicted below the lanes) were added as competitor, as indicated above the lanes. Position of complexes T', T, L and S are indicated by the arrows. **(B) **EMSAs were performed on PA2 probe of *pax6b *(PA2b) using nuclear extract of pancreatic TU6 cells. 10 ng of unlabeled oligonucleotides PA2b (sequence depicted below the lanes), PA1b, UE-A or mutated UE-A were added as competitor. Position of complexes L and S are indicated by the arrows. Abbreviations: **PBX1a and PREP1**, *in vitro*-translated PBX1a and PREP1 proteins; **PDX1**, recombinant PDX1; **PDX1 Ab**, PDX1 antiserum; **PBXL. Ab**, antiserum recognizing specifically the long PBX isoforms (PBX1a, PBX2, and PBX3a) (Santa Cruz Biotechnology); **PREP1 Ab**, PREP1 antibody; **nsp**., nonspecific complex, **Ret. lysate**, reticulocyte lysate.

As regions A and C are involved in pancreatic expression, we looked for pancreatic transcription factors binding to these regulatory regions. A detailed sequence comparison of the A regions of different vertebrate *Pax6 *genes revealed two highly conserved motifs, named PA1 and PA2 (see Fig. 2B). The PA1 motif, which is divergent only in the zebrafish *pax6a *gene, contains the motif ATCAATCA, exactly matching the consensus binding site of PBX factors [52-54]. The PA2 motif, present in the zebrafish *pax6a *gene but with a mismatch at the 3' end and one at the 5' ends, also contains a related-PBX consensus binding site TCAATC. PBX factors are homeoproteins belonging to the PBC TALE class, that have been shown to form heterodimeric complexes with homeproteins of the HM-TALE class, the MEIS and PREP factors [55-58]. These dimeric PBX-MEIS/PREP complexes bind specific motifs within enhancers or promoters and can cooperate with HOX-type homeoproteins bound to neighboring elements [56,59]. For example, we have previously shown that in pancreatic cells, the UE-A element of the somatostatin promoter is recognized by a dimeric PBX-PREP complex cooperating with the pancreatic factor PDX1, a HOX-type homeoprotein, bound to the adjacent TSE1 element [7]. This prompted us to investigate whether the PA1 and PA2 motifs of *pax6b *(named PA1b and PA2b) might be recognized by PBX-MEIS/PREP complexes. EMSAs were performed on the PA1b and PA2b elements with nuclear extracts from pancreatic endocrine cell lines (TU6, RIN) (shown for TU6 extract in Fig. 6A, 6B). Incubation of pancreatic nuclear cell extract with PA1b led to the formation of two strong closely migrating complexes named S and L, and two fainter, more slowly migrating bands named T and T'(arrows, lane 1, Fig. 6A). All the complexes were easily displaced by adding a 100-fold molar excess of unlabeled PA1b, demonstrating the high affinity of the nuclear factors for this element (lane 2, Fig. 6A). Addition of unlabeled somatostatin element UE-A, which binds to the PBX-PREP complex, also displaced the binding on the probe, but the UE-A element mutated in the PBX consensus motif was unable to compete (lanes 3 and 4, Fig. 6A). This indicates that the same proteins in the pancreatic extract recognize both *cis*-elements PA1b and UE-A. We also noticed a specific increase in the intensity of complexes T and T' when the amount of pancreatic TU6 cell extract was raised (lanes 5 and 6, Fig. 6A). To determine whether the PBX and PREP1 factors actually bind to these elements, supershift assays were performed with antibodies raised against the PBX and PREP1 homeoproteins. When the pancreatic extracts were incubated with an antibody reacting specifically with the long PBX isoforms (PBX1a, PBX2 and PBX3a) but not with the short isoforms (PBX1b and PBX3b), the formation of the slower migrating complex L and the higher T'complex were specifically blocked (lane 8, Fig. 6A). Likewise, when pancreatic extracts were incubated with PREP1 antibody, the four complexes S, L, T and T'were disrupted (lane 7, Fig. 6A). These results indicate that complexes S, L, T and T' contain PBX and PREP homeoproteins. Furthermore, complexes S and L were observed with nuclear extract from all tested non-pancreatic cell lines (i.e. COS, Hela, HCT116, etc., data not shown), but the T and T' complexes were observed only with extracts from pancreatic cells (TU6 and Rin cell lines) (data not shown). As TALE homeoproteins are cofactors for HOX-type homeoproteins and as PDX1 is a major pancreatic homeoprotein, we tested whether the PDX1 protein might be present in T and/or T'complexes. Addition of an anti-PDX1 antiserum to the pancreatic extract supershifted both T and T'complexes, whereas complexes S and L were not affected (lane 9, Fig. 6A). We next attempted to reconstitute the protein-DNA complexes observed, using *in vitro*-translated PDX1, PBX1a and PREP1 factors. No protein-DNA complex was observed with PBX1a and PREP1 proteins were tested separately (data not shown). However, when PBX1a (long isoform) and PREP1 were added simultaneously, a protein-DNA complex was generated at the same level as the L complex (lane 11, Fig. 6A). The T'-like complex could also be generated by incubating the PA1b probe with recombinant PDX1, PBX1a and PREP1 (lane 12, Fig. 6A). It is noteworthy that PDX1 alone was unable to bind the PA1b element in the absence of PBX and PREP1 factors (lane 13, Fig. 6A). When the corresponding sequence of *pax6a *was tested by EMSA, no binding of PDX1, PBX or PREP proteins was detected, as demonstrated by the lack of competition with PA1b or UE-A element (see additional file 1); conversely, the binding of PDX1, PBX and PREP factors on the PA1b probe was not displaced by adding an excess of unlabelled PA1a (see additional file 2). Thus, all these results show that the conserved PA1 element of the *pax6b *promoter (PA1b) binds a pancreatic trimeric proteins complex comprising the ubiquitous factors PBX, PREP1 and the pancreatic factor PDX1.

When EMSAs were performed with the PA2b probe (Fig. 6B), we also observed formation of complexes S and L due to binding of the PBX-PREP dimer (lane 1, Fig. 6B). Indeed, both the S and L complexes were specifically displaced by unlabelled PA2b, PA1b or UE-A elements (lanes 2, 3 and 5, Fig. 6B) and blocked by the addition of PREP1 antibody (lane 7, Fig. 6B). Furthermore, the L complex was prevented by the antibody recognizing the long forms of PBX proteins (lane 9, Fig. 6B). In these experiments, we never detected any slower migrating T or T'complex, and the S and L complexes were observed with extracts from both pancreatic and non-pancreatic cell lines, in agreement with the ubiquitous expression of PREP1 and of PBX proteins.

### PDX1 and PBX-PREP heterodimer bind two *cis*-elements within the C region of the zebrafish *pax6b *gene

**Figure 7:**
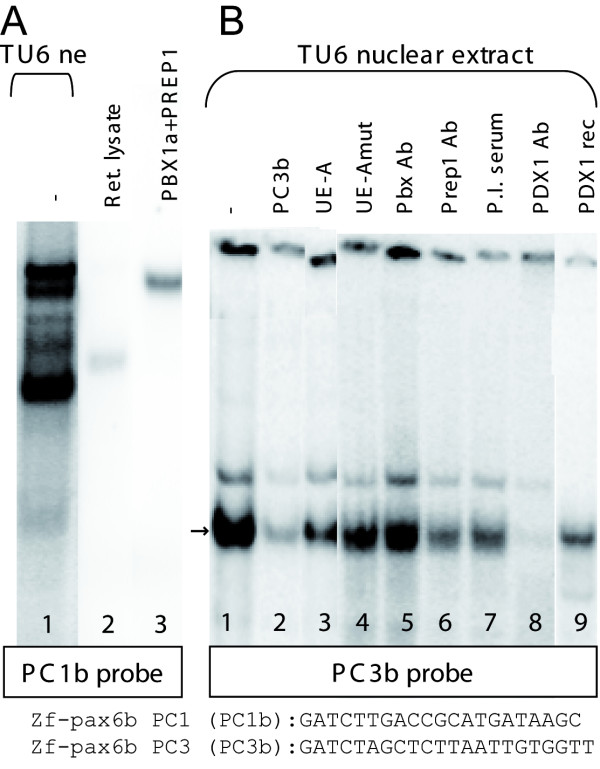
**PBX-PREP dimers bind to the P0-*pax6b *PC1 element and PDX1 binds to PC3b as a monomer**. **(A) **EMSAs were performed on PC1 probe of *pax6b *(PC1b) using nuclear extract of pancreatic TU6 cells. **(B) **EMSAs were performed on PC3 probe of *pax6b *(PC3b) using nuclear extract of pancreatic TU6 cells. 10 ng of unlabeled oligonucleotides PC3b, UE-A and mutated UE-A were added as competitor. Abbreviations: **PBX1a and PREP1**, recombinant PBX1a and PREP1 proteins produced *in vitro *in a reticulocyte extract; **PDX1 Ab**, PDX1 antiserum; **PBXL. Ab**, antiserum recognizing specifically the long PBX isoforms (PBX1a, PBX2, and PBX3a) (Santa Cruz Biotechnology); **PREP1 Ab**, PREP1 antibody PBX1a and PREP1; **P.I. serum**, pre-immune serum.

As region C of the zebrafish *pax6b *gene was able to drive some expression in the pancreas, we looked for transcription factors binding to this element. A sequence comparison of the C regions of various vertebrate *Pax6 *genes, including both zebrafish *pax6 *genes (see Fig. 2B), revealed several motifs well-conserved through evolution. Surprisingly, region C of *pax6a*, displaying no pancreatic activity (see Fig. 5), differs by only 2 nucleotides from the consensus sequence of the other vertebrate *Pax6 *genes (see red stars in Fig. 2B). It is noteworthy that the PC1 element, reported to be required for pancreatic expression and to bind a PBX-PREP dimmer [60], is found in both *pax6a *and *pax6b *and thus cannot explain the differential activity of these genes. In accordance to the reports of Zhang and collaborators, we also observed binding of PBX-PREP dimer on the zebrafish PC1 element of the *pax6b *gene (lanes 1 and 3, fig. 7A). Interestingly, one of the two positions, where the *pax6a *gene diverges from the *pax6b *gene, is located in a conserved motif, named PC3. This element contains a well-conserved TAAT motif present in all vertebrate *Pax6 *genes except chicken *Pax6 *and the zebrafish *pax6a *(see Fig. 2B). This motif is highly similar to the PDX1-binding P element of the insulin promoter [8,61]. When we tested the PC3 element of the zebrafish *pax6b *in EMSA (PC3b) with pancreatic cell extract, we observed formation of one major specific complex (arrow, lane 1, Fig. 7B) which is perturbated by a 100-fold molar excess of unlabeled PC3b, demonstrating the high affinity of the nuclear factor for this element (lane 2, Fig. 7B). No significant competition was observed with unlabeled UE-A or UE-Amut (lanes 3 and 4, Fig. 7B) and no supershift was observed by the PBX and PREP1 antibodies (lanes 5 and 6 fig. 7B). On the other hand, incubation of the extract with anti-PDX1 antibody caused the disappearance of the complex (lane 8, Fig. 7B), and addition of the recombinant PDX1 protein to the PC3b probe was sufficient to reconstitute a complex showing the same mobility (lane 9, Fig. 7B). This demonstrates that the pancreatic homeobox PDX1 factor binds as a monomer to the conserved PC3b element. Importantly, when the corresponding PC3 element of *pax6a *(named PC3a) was tested by EMSA, no binding of PDX1 could be detected (see additional file 3).

### Synergistic activation of the P0-*pax6b *promoter by PBX, PREP1 and PDX1

**Figure 8:**
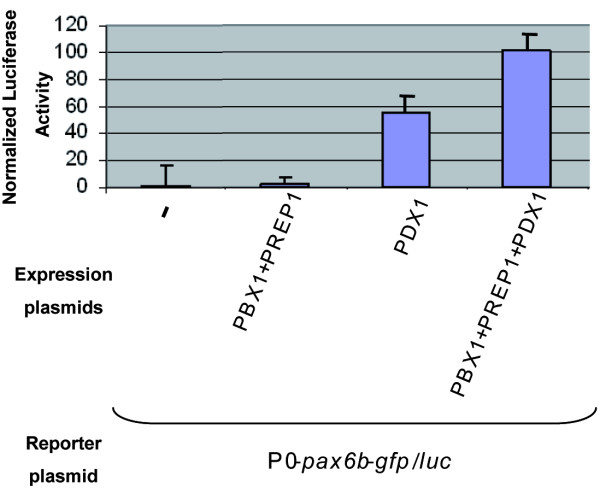
**Synergistic activation of the P0-*pax6b *promoter by PBX1a, PREP1 and PDX1 factors**. HCT116 cells were transfected with the P0*pax6b*-*gfp*/*luc *reporter plasmid. This reporter plasmid was cotransfected with expression vectors for PBX1a, PREP1 and/or PDX1 as indicated. Luciferase activities were normalized to *β*-galactosidase activity generated by the internal control plasmid Rous sarcoma virus-β-galactosidase. Normalized Luc activity obtained in the cell transfected without expression vector was arbitrarily set at 1. The data are means ± S.D. of three transfection experiments, each performed in duplicate.

As several conserved elements of the *pax6b *P0 promoter are bound by PDX1 and/or the PBX-PREP complex, we next investigated how these proteins affect the activity of the P0 promoter. To this end, we generated a reporter plasmid carrying the *pax6b *P0 promoter inserted upstream from a *gfp*/*luciferase *gene. We then tested the activity of this construct by transient cell transfection in the presence of expression vectors for PBX1a, PREP1 and PDX1. The activity of the P0-*pax6b *promoter is very low in non pancreatic cells, such as the intestinal carcinoma cell line HCT116 (Fig. 8). While co-transfection of both PBX1a and PREP1 expression vectors had no profound effect, co-transfection of the *Pdx1*-expressing vector alone was sufficient to enhance the *pax6b *P0 promoter activity significantly, and this activity was increased still further in the presence of PBX1a and PREP1 to reach a global activation of about 100-fold.

## Discussion

PAX6 is an essential transcription factor that plays a role in several developmental processes, such as endocrine pancreatic cell differentiation and eye morphogenesis. Identification of *cis*-regulatory elements and *trans*-acting factors driving *Pax6 *expression will contribute to our understanding of the mechanisms controlling the development of these tissues. Here we have analyzed expression of the two zebrafish paralogs *pax6a *and *pax6b*, showing that only *pax6b *is expressed in the pancreas. Our results of genomic sequence comparisons, transient and stable transgenesis assays in zebrafish, and *in vitro *studies, show that this differential expression is due to the regulatory regions A and C, located upstream from the P0 promoter. These regulatory regions contain two key pancreatic elements, PA1 and PC3, present in the *pax6b *gene and not (or less conserved) in the *pax6a *gene. We show that the PA1 element is specifically bound by a heterotrimeric complex composed of the homeoproteins PDX1, PBX and PREP, whereas the PC3 element is recognized by PDX1 alone.

### Pancreatic expression of the zebrafish *pax6b *gene relies on the combined action of the conserved regions A and C

Two different groups have previously shown by mouse transgenesis that the regulatory elements targeting expression of *Pax6 *to pancreatic endocrine cells are located upstream from the P0 promoter [31,32]. Yet on the basis of results obtained with 5' deletions within this promoter, they disagree as to the precise location of these pancreatic elements: whereas Kammandel et al. highlight an essential element at 3.3 kb upstream from exon 0 [32], the group of Maas locates the element 1.9 kb upstream from this same exon 0 [31,42,60]. To resolve this ambiguity, we have carried out detailed comparisons of genomic sequences upstream from the P0 promoter among various vertebrates. We show that three regions (A, B and C) are conserved from teleosts to mammals. Region A, corresponding to the pancreatic element identified by Kammandel and co-workers, contains two motifs (PA1 and PA2) that are strictly conserved in all vertebrate *Pax6 *genes examined, except in the zebrafish *pax6a *gene, where the PA1 motif is missing. In contrast, both region B and region C are well conserved in the two zebrafish *pax6 *genes, region B overlapping with the lens-specific enhancer [40] and region C corresponding with the pancreatic element identified by Xu [31].

The results of our study indicate that the pancreatic expression actually relies on the combined action of the two regulatory regions A and C, identified in the two previous studies. This is supported by at least three observations: i) deletion of region A significantly decreases pancreatic expression but does not abolish it completely; ii) region C is sufficient to drive weaker but detectable pancreatic expression, but deletion of that region, in the context of *pax6b *P0 promoter, does not abolish pancreatic expression in transient assays; iii) the *pax6b *P0 promoter, deleted of region B and retaining regions A and C, is strongly active in the pancreas in both mosaic (transient assays) and stable transgenic embryos. Our results clearly show that region C is also important for the overall activity of the *pax6b *P0 promoter, since deletion of region C completely abolishes P0 promoter activity in all tissues in the three distinct stable transgenic lines obtained, and since it causes a major reduction of the total number of DSRED/GFP-expressing cells in transient transgenic embryos. The stronger effect of region C in stable transgenics may be due to stronger epigenetic regulation processes (chromatin structure and/or methylation) (reviewed in [62]). Indeed, the expression being analysed 2 days, in case of transient transgenics, or more than 100 days in case of stable transgenics (and in the next generations), after injection of the transgenes, we can assume that the epigenetic processes controlling transgene expression are more stringent in the stable transgenic lines. Very recently, another study reported the differential expression of the two zebrafish *pax6 *genes and analysed the cause of this differential expression [25]. While they used a similar strategy based on the transient expression of *pax6b*P0:*gfp *constructs in mosaic transgenic embryos, these authors pointed up the importance of the conserved region (AB) for the pancreatic expression, but not of region C. The reason of this discrepancy is unclear but could be due to the different designs of promoter deletions and swappings, and/or to a different level of assay sensitivity. In the present study, we analyse in more details the sequence motifs divergent in the two zebrafish promoters and we succeed to identify the transcription factors binding to these motifs.

### Binding of the PDX1, PBX and PREP proteins to *cis*-elements within regions A and C

We show here that the two most conserved elements of region A, PA1 and PA2, are bound by the TALE class homeoproteins PBX and PREP. Furthermore, the PA1 motif is recognized by a trimeric complex composed of PBX, PREP and the pancreatic factor PDX1. These three transcription factors cooperate to stimulate the activity of *pax6b *P0 promoter in transfected cells (see Fig. 8). The PA1 motif is not conserved in the *pax6a *gene, and the pancreatic trimeric complex is unable to bind to region A of *pax6a*. It is noteworthy that PDX1 does not bind the PA1b element at all when tested alone. It absolutely requires the cooperative binding of the dimeric complex PBX-PREP. In contrast, although the UE-A element of the somatostatin gene contains the same motif, ATCAATCA, this UE-A site is recognized by a PBX-PREP heterodimer but not a PDX1-PBX-PREP heterotrimer [7]. This reveals the importance of the sequences flanking PA1b in formation of the trimeric complex.

Zhang and coworkers have reported the binding of a PBX-PREP dimer on another conserved sequence of the *Pax6 *P0 promoter, located in region C [60]. They found mutation of that particular element to strongly affect the activity of the P0 promoter. In agreement with their results, we also detected binding of a PBX-PREP dimer to the homologous zebrafish sequence. Yet this element, here referred to as PC1, is also present in the zebrafish *pax6a *gene, which is not expressed in the pancreas. Furthermore, it is bound by a ubiquitous PBX-PREP complex and not by a pancreas-specific complex. Thus, the pancreatic regulatory activity of region C cannot be attributed to the PC1 site per se. On the other hand, we demonstrate binding of the pancreatic factor PDX1 to the PC3 element of the *pax6b *gene, while the corresponding sequence in the paralogous zebrafish *pax6a *gene is mutated and cannot bind to PDX1. This provides a good explanation of the differential activity of the C regions between the two zebrafish *pax6 *genes. The function of PC3 site could be further tested by introducing point mutations in the PC3b site within the pax6b(C)-c-fos:gfp fusion construct. The PC3 element is well conserved through evolution and was found in all analysed vertebrate *Pax6 *genes (human, mouse, rat, bovine, cat, frog, fugu, tetraodon, stickelback *Pax6 *and zebrafish *pax6b*) except in the zebrafish *pax6a *gene and in the chicken *Pax6 *gene (Fig. 2 and data not shown). The absence of the PC3 element in the chicken *Pax6 *gene is quite surprising, as *Pax6 *is expressed in pancreas in chick embryos [63]. It is nevertheless possible that another PC3-like element, elsewhere in the chicken P0 promoter, compensates for the absence of region C and acts cooperatively with the elements of region A.

### Regulatory elements of zebrafish *pax6b *driving expression in the retina, in enteroendocrine cells, and in the telencephalon

While the zebrafish *pax6b *P0 promoter drives reporter expression in pancreatic endocrine cells in agreement with data reported on the mouse *Pax6 *P0 promoter, striking differences are found as regards expression in the other tissues. Firstly, we never detected any GFP/DSRED expression in the lens tissue of the six stable zebrafish transgenic lines, whereas all studies on the mouse P0 promoter have demonstrated a lens-specific enhancer located 3.5 kb upstream from the mouse P0 promoter (corresponding to the conserved region B) [40]. Our study indicates that the region B of zebrafish *pax6b *is required for expression, not in the lens, but in the retina of zebrafish embryos. Our data are in agreement with results obtained by Woolfe and collaborators showing that the homologous region B of the zebrafish *pax6a *is suffcient to target expression also in the retina [30]. Secondly, Xu and collaborators report that the conserved C region, enables the mouse *Pax6 *P0 promoter to drive reporter expression in some progenitor cells within the retina [31]. Our transgenic zebrafish do not show any GFP/DSRED expression in retinal progenitor cells, but only at later stage, in differentiated retinal neurons (i.e. ganglion cells).

Our study also demonstrates that the zebrafish *pax6b *P0 promoter drives expression in the telencephalon and in enteroendocrine cells, whereas no such expression has been reported with the mouse P0 promoter. Such differences are quite surprising and puzzling. The activity of the mouse *Pax6 *P0 promoter in the enteroendocrine cells of transgenic mice has probably been missed, as the developing gut produces a non specific *LacZ *background staining likely to mask expression in the scattered enteroendocrine cells. Thus, it would be interesting to re-examine the developing gut of the *Pax6*:*LacZ *transgenic mice by other approaches (i.e. by *in situ *hybridization). Our study identifies, for the first time, a regulatory region in the *Pax6 *locus driving expression in enteroendocrine cells; this enhancer requires the conserved B and C regions.

Finally, there are no reports of *LacZ *reporter expression in the telencephalon with the mouse *Pax6 *P0 promoter, in contrast to our data on the zebrafish *pax6b *P0 promoter. Telencephalon-specific enhancers have been detected within the murine *Pax6 *locus but they are located upstream from the P1 promoter, in intron 7 and far downstream from the gene [32,64]. Translocation of such enhancers within the zebrafish *pax6b *promoter is very unlikely, as bioinformatic analyses have failed to reveal any significant sequence similarity to these enhancers. Furthermore, our data of stable transgenic fish show that expression in the telencephalon requires all the conserved regions A, B and C. More deletion constructs will be necessary to delineate more precisely the regulatory elements controlling *pax6b *transcription in the telencephalon.

## Conclusion

In conclusion, this study shows that the two zebrafish *pax6 *genes are differentially expressed and that this is attributable to divergence in two conserved regulatory cis-elements binding the pancreatic factor PDX1. We also demonstrate that the zebrafish *pax6b *P0 promoter targets expression not only to the endocrine pancreas, but also to the retina, the telencephalon, the diencephalon and to enteroendocrine cells. Further interspecies sequence comparisons and analysis of additional transgenic constructs will help to delineate precisely the regulatory elements targeting these different tissues.

## Methods

### Sequence comparison

Sequence comparisons are based on the seven *pax6b *transcripts described on Vega website [65]; *pax6b *Vega gene: OTTDARG00000018846, and on the fourteen *pax6a *transcripts; *pax6a *Vega gene: OTTDARG00000018854. The conserved regulatory regions were found by performing dot-plot comparison of the mouse *Pax6 *and zebrafish *pax6a *and *pax6b *genes using the MEGALIGN software of the DNAStar package. Parameters were set to a minimum match of 60% over a window of 30 nucleotides. Multiple alignments of the three conserved regions upstream the vertebrate *Pax6 *P0 promoters (fig. 2B) were then performed using the AlignX software of the Vector NTI 9.0 Advance software package.

### Cloning of the zebrafish *pax6b *P0 promoter and construction of transgenes

A PAC clone spanning the zebrafish *pax6b *gene was isolated by PCR screening PAC library #706 [66]using the *pax6b *cDNA primers BP249 and BP251. *EcoR*I fragments of the identified positive PAC were then subcloned in pUC13 plasmids. A clone containing 7 kb of the *pax6b *gene and possessing exon 0, part of intron 0, the P0 promoter and the upstream conserved element was then identified by PCR with primers BP311 and BP313, corresponding to the sequences conserved between the mouse, human and fugu *Pax6 *P0 promoters (described as region B by [32]). The insert was sequenced on both strands using the EZ::TN<TET> Insertion kit (Epicentre Technologies). In order to clone the *pax6b *P0 promoter upstream from the *gfp *coding region, a *BamH*I site was created by PCR within exon 0, 120 bp downstream from the transcription start site. Then, the 3850 bp *EcoR*I-*BamH*I DNA fragment was inserted upstream from the *gfp *sequence in the pG1 vector (gift of Chi-Bien Chien and Darren Gilmour) or upstream the *dsred *sequence in the pSX vector containing *Sce*-I meganuclease sites on each side of the transgene (gift of Wolfgang Driever). To obtain the deletion constructs, the P0-*pax6b*-*dsred*-pSX was digested by *Acc*I, *Bst11071*I and *Bcl*I, and *EcoN*I for deletion of enhancer A, B and C respectively, and then re-ligated.

The 3850 bp *EcoR*I-*BamH*I DNA fragment was also cloned in a "Gateway" pCR8/GW/TOPO entry vector (Invitrogen). This plasmid was then recombined in the pDestTol2pA destination vector with two other entry vectors, the P5E-MCS and the P3E-egfp (gifts of Chi-Bin Chien and K. Kawakami), by a triple recombination using the LR Clonase enzyme (Invitrogen) as described by Kwan et al. [67]. Deletion of element A or C was performed by digesting the P0-*pax6b*-*egfp*Tol2 pA with *BstZ17*I and *Sal*I, and *BspM*I respectively.

To clone the regulatory elements ABC, AB and C of *pax6a *and *pax6b *in front of the heterologous *cfos *promoter driving GFP, these elements were first amplified from the P0-*pax6b*-*egfp*Tol2 pA plasmid (for *pax6b*) or from the genomic DNA (for *pax6a*). The ensuing PCR products were then inserted into an entry vector (pCR8/GW/TOPO). The resulting constructs were recombined by simple LR recombination in the destination vector pGW_*cfos*_egfp (gift of S. Fisher) as described by Fisher et al. [68,69]. The primers used to amplify the regulatory elements are as follow:

ABC *pax6*a: BP568/563

AB *pax6a*: BP568/BP570

C *pax6b*: BP571/BP563

ABC *pax6b*: BP559/560

AB *pax6b*: BP559/566

C *pax6b*: BP567/BP560

For further primer codes, see Table 1.

**Table 1:** Primer codes.

Primer code	Sequence
BP249	CGGGCTCCATCCGACCG
BP251	CAGGTTGCGTAGCACTCGG
BP311	CATTATTGTTGCCAGCACGAAGCATC
BP313	AGTAGACAGGCACGTCCTGGATGT
BP559	GACAGACAGATAGACAGAAAGATAG
BP560	CAGGATGTGGAGTAAAGGTGAAGC
BP563	TTTCCTAGCAGCTTTATTTTTATGA
BP566	TGTCGTCACAGTTTTCTTTCAGA
BP567	TGAGGGGGAGAGAGACACATAG
BP568	AATCAGTCAAAACGAGGCTACC
BP570	AAAATCATGACGGCCAGTTT
BP571	CTTCCCTTGCTTTTCCCTCT

### Microinjection and generation of P0-*pax6b:gfp/dsred *transgenic zebrafish

To generate the transgenic fish, three methods were used. The first one consists in injecting 500 ng of the linearized *EcoR*I-*Not*I fragment from P0-*pax6b*:*gfp *pG1 plasmid into the cytoplasm of fertilized eggs. For *Sce*-I mediated transgenesis, circular P0-*pax6b*/*dsred*-pSX plasmid was injected as described by Thermes et al[50]. For Tol2-mediated transgenesis, circular plasmid was injected as described by Kawakami et al [51].

The injected embryos were incubated at 28°C and GFP/DSRED expression was observed between 24 and 75 hpf using a Leica DC500 photocamera. Pictures were processed with Adobe Photoshop software. For generation of stable transgenic lines, GFP/DSRED-positive embryos were raised to sexual maturity. Transgenic founders were identified by crossing and observation of F1 embryos with an epifluorescence stereomicroscope. Transgenic founders harboring C-deleted construct were identified by performing PCR on genomic DNA extracted from F1 embryos.

### Single and double fluorescent whole mount *in situ *hybridization on zebrafish embryos

Single hybridizations and detection were carried out as previously described [70]. Anti-sense RNA probe were prepared by transcribing a linearized cDNA clone with T7 polymerase using digoxigenin mix (Roche). The probes used in the single hybridizations were *pax6a *and *pax6b *[44]. Double fluorescent hybridizations were performed as described by Mavropoulos et al. [71]. Briefly, zebrafish embryos were incubated in 2% H_2_O_2 _for 60 min for endogenous peroxydase inactivation, just prior to proteinaseK treatment. For hybridization, antisense probes were prepared using digoxigenin labeling mix (Roche) or DNP-11-UTP ribonucleotides (TSAi Plus system, Perkin Elmer). The probes used were: *pdx1 *and *preproinsulin *[72], *glucagon *[73], *neuroD *[74], *somatostatin2 *[75] and *ghrelin *(NCBI: AL918922). The embryos were blocked in 100 mM Tris-HCl pH 7.5, 150 mM NaCl (TNT buffer) with 0.5% Blocking Reagent (Perkin Elmer). For detection, we used pre-absorbed HRP-coupled antidigoxigenin (Roche) or HRP-coupled anti-DNP antibodies (Perkin Elmer). The embryos were then extensively washed in TNT buffer. Revelation was performed by incubating embryos for 60 min in tyramide-FITC and tyramide-Cy3 prepared according to Peter Vize's protocol [76] at a final dilution of 1/50 in 1× Amplification Reagent (Perkin Elmer). Embryos were then stored in TNT buffer.

### Confocal imaging

Confocal imaging was performed by using a LeicaTCS SP2 inverted confocal laser microscope (Leica Microsystems, Germany). Digitized images were acquired using a 10× (NA 0.4) Plan-Apo waterimmersionobjective at 1024 × 1024 pixel resolution. For multicolor imaging, GFP was visualized by using an excitation wavelength of 488 nm and the emission light was dispersed and recorded at 500 to 535 nm. DSRED was detected by using an excitation wavelength of 543 and the fluorescence emission was dispersed and recorded at 560 to 650 nm. The acquisition was set up to avoid any cross-talk of the two fluorescence emissions. Series of optical sections were carried out toanalyze the spatial distribution of fluorescence, and for each embryo, they were recorded with a Z-step ranging between 0.5 and 1.0 Am. Image processing, including background subtraction and projection of Z-stacks, was performed with Leica software (version 2.5). Captured images were exported as TIFF format files and further processed using Adobe Photoshop CS3.

### Electrophoretic mobility shift assays (EMSAs)

EMSAs were carried out exactly as described previously [77]. Briefly, 2 μg of nuclear extract prepared as described by Schreiber et al. [78]or 1 μl of *in vitro *translated protein was incubated with 0,1 ng of a double-stranded oligonucleotide (^32^P-labeled using Klenow polymerase) in presence of 1 μg of poly [d(I-C)]. In supershift experiments, the nuclear cell extracts were preincubated with 1 μl of antiserum (rabbit IgG) for 15 min at room temperature before adding the probe. Antibodies used are PRP-1 (N-15) #sc-6245 and PBX1/2/3 (C-20) #sc-888 (Santa Cruz Biotechnology, Inc.). PDX1 (STF-1) antiserum used is raised against a C-terminal PDX1 polypeptide (amino acids 216–283), as described in Peers et al. [77]. In competition experiments, the cold oligonucleotides were mixed with the probe before addition of the nuclear extract. The sequences of the oligonucleotides (Eurogentec, Liège, Belgium) are:

UE-A: GATCTTCTTTGATTGATTTTGC,

UE-Amut:GATCTTCTTTCCTTCCTTTTGC,

PA1b: GATCGCATCACGATCAATCACAGCGCT,

PA1a: GATCTAATTTTGTTGTACCATGATATCG,

PA2b: GATCTGGCAGCTGTCAATCATTTC,

PA2a: GATCCTGGGAGGCAGATGTCATTATTCG,

PB1b: GATCTACTCAGGCTTGTCACATTGAGGT,

PC1b: GATCTTGACCGCATGATAAGC,

PC3a: GATCGGGGTCTAAACGGTGCAG

PC3b: GATCTAGCTCTTAATTGTGGTT

The Flag-PBX1a and PREP1 proteins were produced *in vitro *using the Promega TNT transcription-translation system, according to the protocol of the manufacturer. PDX1 protein was expressed in *Escherichia *coli using the pGEX3X vector as described previously [77].

### Cell transfection experiments

The reporter plasmid P_0_-*pax6b*-pSX:*gfp*/*luc *used in transient transfection experiments was constructed by digesting the P0-*pax6b*-pSX vector with *Nco*I/*Xba*I to remove the *dsred *coding sequence. In parallel, the *gfp*/*luc *coding sequence was removed from the pGCV plasmid [79] by *Nco*I/*Xba*I digestion and re-ligated in the pSX vector. HCT116 human colon carcinoma cells were grown in RPMI 1640 medium supplemented with 10% of fetal calf serum in 175 cm^3 ^dishes. Transient transfection experiments were performed in HCT116 cells using the Lipofectamine Plus™ reagent (Invitrogen). Cells transfected with 0,8 μg of the reporter plasmid (P0-*pax6b*-pSX:*gfp*/*luc*), 40 to 80 ng of expression vector and 100 ng of *Rous *sarcoma virus-*β*-galactosidase plasmid, used as an internal control, were harversted, lysed, and assayed for the luciferase and *β*-galactosidase activities. Luciferase activities were normalized to *β*-galactosidase activity in each cell extract.

## Authors' contributions

FMD carried out all the molecular genetic studies, participated in the sequence alignment and wrote the manuscript. VP carried out part of the *Sce*-I transgenic assays and part of the EMSAs. ND participated in the initial sequence alignments and the generation of the first GFP transgenic fish. IM took part in the zebrafish cross sections and helped to draft the manuscript. MLV conceived Tol2-mediated transgenesis experiments and helped to draft the manuscript. PM performed confocal analysis. FB took part in the early concept of the study. JAM helped in coordination of the study BP conceived the study and participated in its design and coordination and helped to draft the manuscript. All authors read and approved the final manuscript.

## Supplementary Material

Additional file 1 **No PBX-PREP-PDX1 complex is observed on homologous *pax6a *PA1 sequence**. When we tested the homologous pax6a PA1 sequence (named PA1a), incubation with nuclear extract led to the formation of four major complexes (arrows, lane 1) which were displaced by adding a 100-fold molar excess of unlabeled PA1a (lane 2). Addition of unlabelled PA1b and somatostatin element UE-A, which bind PBX-PREP-PDX1 and PBX-PREP complex, respectively, do not displace the binding on the probe (lanes 3 and 4). This indicates that the proteins in the pancreatic extract which recognize the two cis-elements PA1b and UE-A do not recognize the homologous pax6a element. Click here for file

Additional file 2 **Unlabeled PA1 of *pax6a *does not compete with PA1 of *pax6b***. To further demonstrate that PDX1 is unable to bind to PA1a, this sequence was used as competitor on PA1b probe. No competition was observed, suggesting that PDX1 binds specifically PA1 of *pax6b *and not PA1a. Click here for file

Additional file 3 **PDX1 binds to PC3b and not to PC3a**. EMSAs were performed on PC3 probes of pax6a (PC3a) and *pax6b *(PC3b) using nuclear extract of pancreatic TU6 cells. 10 ng of unlabeled oligonucleotides PC3a (lanes 2, 6), and PC3b (lanes 3, 7) were added as competitors. Abbreviations: **PBX1a and PREP1**, recombinant PBX1a and PREP1 proteins produced *in vitro *in a reticulocyte extract; **PDX1 rec**, PDX1 recombinant. Click here for file

## References

[B1] Jensen J (2004). Gene regulatory factors in pancreatic development. Dev Dyn.

[B2] Murtaugh LC (2007). Pancreas and beta-cell development: from the actual to the possible. Development.

[B3] Ahlgren U, Pfaff SL, Jessell TM, Edlund T, Edlund H (1997). Independent requirement for ISL1 in formation of pancreatic mesenchyme and islet cells. Nature.

[B4] Jonsson J, Carlsson L, Edlund T, Edlund H (1994). Insulin-promoter-factor 1 is required for pancreas development in mice. Nature.

[B5] Offield MF, Jetton TL, Labosky PA, Ray M, Stein RW, Magnuson MA, Hogan BL, Wright CV (1996). PDX-1 is required for pancreatic outgrowth and differentiation of the rostral duodenum. Development.

[B6] Dutta S, Gannon M, Peers B, Wright C, Bonner-Weir S, Montminy M (2001). PDX:PBX complexes are required for normal proliferation of pancreatic cells during development. Proc Natl Acad Sci U S A.

[B7] Goudet G, Delhalle S, Biemar F, Martial JA, Peers B (1999). Functional and cooperative interactions between the homeodomain PDX1, Pbx, and Prep1 factors on the somatostatin promoter. J Biol Chem.

[B8] Peers B, Sharma S, Johnson T, Kamps M, Montminy M (1995). The pancreatic islet factor STF-1 binds cooperatively with Pbx to a regulatory element in the somatostatin promoter: importance of the FPWMK motif and of the homeodomain. Mol Cell Biol.

[B9] Swift GH, Liu Y, Rose SD, Bischof LJ, Steelman S, Buchberg AM, Wright CV, MacDonald RJ (1998). An endocrine-exocrine switch in the activity of the pancreatic homeodomain protein PDX1 through formation of a trimeric complex with PBX1b and MRG1 (MEIS2). Mol Cell Biol.

[B10] Edlund H (2002). Pancreatic organogenesis--developmental mechanisms and implications for therapy. Nat Rev Genet.

[B11] Gradwohl G, Dierich A, LeMeur M, Guillemot F (2000). neurogenin3 is required for the development of the four endocrine cell lineages of the pancreas. Proc Natl Acad Sci U S A.

[B12] Huang HP, Tsai MJ (2000). Transcription factors involved in pancreatic islet development. J Biomed Sci.

[B13] Mellitzer G, Bonne S, Luco RF, Van De Casteele M, Lenne-Samuel N, Collombat P, Mansouri A, Lee J, Lan M, Pipeleers D, Nielsen FC, Ferrer J, Gradwohl G, Heimberg H (2006). IA1 is NGN3-dependent and essential for differentiation of the endocrine pancreas. Embo J.

[B14] Collombat P, Mansouri A, Hecksher-Sorensen J, Serup P, Krull J, Gradwohl G, Gruss P (2003). Opposing actions of Arx and Pax4 in endocrine pancreas development. Genes Dev.

[B15] Inoue H, Rudnick A, German MS, Veile R, Donis-Keller H, Permutt MA (1997). Isolation, characterization, and chromosomal mapping of the human Nkx6.1 gene (NKX6A), a new pancreatic islet homeobox gene. Genomics.

[B16] Sosa-Pineda B, Chowdhury K, Torres M, Oliver G, Gruss P (1997). The Pax4 gene is essential for differentiation of insulin-producing beta cells in the mammalian pancreas. Nature.

[B17] St-Onge L, Sosa-Pineda B, Chowdhury K, Mansouri A, Gruss P (1997). Pax6 is required for differentiation of glucagon-producing alpha-cells in mouse pancreas. Nature.

[B18] Sussel L, Kalamaras J, Hartigan-O'Connor DJ, Meneses JJ, Pedersen RA, Rubenstein JL, German MS (1998). Mice lacking the homeodomain transcription factor Nkx2.2 have diabetes due to arrested differentiation of pancreatic beta cells. Development.

[B19] Heller RS, Stoffers DA, Liu A, Schedl A, Crenshaw EB, Madsen OD, Serup P (2004). The role of Brn4/Pou3f4 and Pax6 in forming the pancreatic glucagon cell identity. Dev Biol.

[B20] Sander M, Sussel L, Conners J, Scheel D, Kalamaras J, Dela Cruz F, Schwitzgebel V, Hayes-Jordan A, German M (2000). Homeobox gene Nkx6.1 lies downstream of Nkx2.2 in the major pathway of beta-cell formation in the pancreas. Development.

[B21] Callaerts P, Halder G, Gehring WJ (1997). PAX-6 in development and evolution. Annu Rev Neurosci.

[B22] Kioussi C, Gruss P (1994). Differential induction of Pax genes by NGF and BDNF in cerebellar primary cultures. J Cell Biol.

[B23] Mansouri A (1998). The role of Pax3 and Pax7 in development and cancer. Crit Rev Oncog.

[B24] Walther C, Gruss P (1991). Pax-6, a murine paired box gene, is expressed in the developing CNS. Development.

[B25] Kleinjan DA, Bancewicz RM, Gautier P, Dahm R, Schonthaler HB, Damante G, Seawright A, Hever AM, Yeyati PL, van Heyningen V, Coutinho P (2008). Subfunctionalization of Duplicated Zebrafish pax6 Genes by cis-Regulatory Divergence. PLoS Genet.

[B26] Lakowski J, Majumder A, Lauderdale JD (2007). Mechanisms controlling Pax6 isoform expression in the retina have been conserved between teleosts and mammals. Dev Biol.

[B27] Morgan R (2004). Pax6 is a direct, positively regulated target of the circadian gene Clock. Dev Dyn.

[B28] Kleinjan DA, Seawright A, Mella S, Carr CB, Tyas DA, Simpson TI, Mason JO, Price DJ, van Heyningen V (2006). Long-range downstream enhancers are essential for Pax6 expression. Dev Biol.

[B29] Plaza S, Saule S, Dozier C (1999). High conservation of cis-regulatory elements between quail and human for the Pax-6 gene. Dev Genes Evol.

[B30] Woolfe A, Goodson M, Goode DK, Snell P, McEwen GK, Vavouri T, Smith SF, North P, Callaway H, Kelly K, Walter K, Abnizova I, Gilks W, Edwards YJ, Cooke JE, Elgar G (2005). Highly conserved non-coding sequences are associated with vertebrate development. PLoS Biol.

[B31] Xu PX, Zhang X, Heaney S, Yoon A, Michelson AM, Maas RL (1999). Regulation of Pax6 expression is conserved between mice and flies. Development.

[B32] Kammandel B, Chowdhury K, Stoykova A, Aparicio S, Brenner S, Gruss P (1999). Distinct cis-essential modules direct the time-space pattern of the Pax6 gene activity. Dev Biol.

[B33] Plaza S, Dozier C, Langlois MC, Saule S (1995). Identification and characterization of a neuroretina-specific enhancer element in the quail Pax-6 (Pax-QNR) gene. Mol Cell Biol.

[B34] Plaza S, Turque N, Dozier C, Bailly M, Saule S (1995). C-Myb acts as transcriptional activator of the quail PAX6 (PAX-QNR) promoter through two different mechanisms. Oncogene.

[B35] Griffin C, Kleinjan DA, Doe B, van Heyningen V (2002). New 3' elements control Pax6 expression in the developing pretectum, neural retina and olfactory region. Mech Dev.

[B36] Kleinjan DA, Seawright A, Schedl A, Quinlan RA, Danes S, van Heyningen V (2001). Aniridia-associated translocations, DNase hypersensitivity, sequence comparison and transgenic analysis redefine the functional domain of PAX6. Hum Mol Genet.

[B37] Dimanlig PV, Faber SC, Auerbach W, Makarenkova HP, Lang RA (2001). The upstream ectoderm enhancer in Pax6 has an important role in lens induction. Development.

[B38] Purcell P, Oliver G, Mardon G, Donner AL, Maas RL (2005). Pax6-dependence of Six3, Eya1 and Dach1 expression during lens and nasal placode induction. Gene Expr Patterns.

[B39] Williams SC, Altmann CR, Chow RL, Hemmati-Brivanlou A, Lang RA (1998). A highly conserved lens transcriptional control element from the Pax-6 gene. Mech Dev.

[B40] Zhang X, Friedman A, Heaney S, Purcell P, Maas RL (2002). Meis homeoproteins directly regulate Pax6 during vertebrate lens morphogenesis. Genes Dev.

[B41] Donner AL, Episkopou V, Maas RL (2007). Sox2 and Pou2f1 interact to control lens and olfactory placode development. Dev Biol.

[B42] Zhang X, Heaney S, Maas RL (2003). Cre-loxp fate-mapping of Pax6 enhancer active retinal and pancreatic progenitors. Genesis.

[B43] Nornes S, Clarkson M, Mikkola I, Pedersen M, Bardsley A, Martinez JP, Krauss S, Johansen T (1998). Zebrafish contains two pax6 genes involved in eye development. Mech Dev.

[B44] Krauss S, Johansen T, Korzh V, Moens U, Ericson JU, Fjose A (1991). Zebrafish pax[zf-a]: a paired box-containing gene expressed in the neural tube. Embo J.

[B45] Puschel AW, Gruss P, Westerfield M (1992). Sequence and expression pattern of pax-6 are highly conserved between zebrafish and mice. Development.

[B46] Biemar F, Argenton F, Schmidtke R, Epperlein S, Peers B, Driever W (2001). Pancreas development in zebrafish: early dispersed appearance of endocrine hormone expressing cells and their convergence to form the definitive islet. Dev Biol.

[B47] Krauss S, Johansen T, Korzh V, Fjose A (1991). Expression pattern of zebrafish pax genes suggests a role in early brain regionalization. Nature.

[B48] Ton CC, Hirvonen H, Miwa H, Weil MM, Monaghan P, Jordan T, van Heyningen V, Hastie ND, Meijers-Heijboer H, Drechsler M (1991). Positional cloning and characterization of a paired box- and homeobox-containing gene from the aniridia region. Cell.

[B49] Malicki J (2004). Cell fate decisions and patterning in the vertebrate retina: the importance of timing, asymmetry, polarity and waves. Curr Opin Neurobiol.

[B50] Thermes V, Grabher C, Ristoratore F, Bourrat F, Choulika A, Wittbrodt J, Joly JS (2002). I-SceI meganuclease mediates highly efficient transgenesis in fish. Mech Dev.

[B51] Kawakami K (2005). Transposon tools and methods in zebrafish. Dev Dyn.

[B52] LeBrun DP, Cleary ML (1994). Fusion with E2A alters the transcriptional properties of the homeodomain protein PBX1 in t(1;19) leukemias. Oncogene.

[B53] Lu Q, Wright DD, Kamps MP (1994). Fusion with E2A converts the Pbx1 homeodomain protein into a constitutive transcriptional activator in human leukemias carrying the t(1;19) translocation. Mol Cell Biol.

[B54] van Dijk MA, Peltenburg LT, Murre C (1995). Hox gene products modulate the DNA binding activity of Pbx1 and Pbx2. Mech Dev.

[B55] Abu-Shaar M, Ryoo HD, Mann RS (1999). Control of the nuclear localization of Extradenticle by competing nuclear import and export signals. Genes Dev.

[B56] Berthelsen J, Kilstrup-Nielsen C, Blasi F, Mavilio F, Zappavigna V (1999). The subcellular localization of PBX1 and EXD proteins depends on nuclear import and export signals and is modulated by association with PREP1 and HTH. Genes Dev.

[B57] Mercader N, Leonardo E, Azpiazu N, Serrano A, Morata G, Martinez C, Torres M (1999). Conserved regulation of proximodistal limb axis development by Meis1/Hth. Nature.

[B58] Rieckhof GE, Casares F, Ryoo HD, Abu-Shaar M, Mann RS (1997). Nuclear translocation of extradenticle requires homothorax, which encodes an extradenticle-related homeodomain protein. Cell.

[B59] Nakamura T, Jenkins NA, Copeland NG (1996). Identification of a new family of Pbx-related homeobox genes. Oncogene.

[B60] Zhang X, Rowan S, Yue Y, Heaney S, Pan Y, Brendolan A, Selleri L, Maas RL (2006). Pax6 is regulated by Meis and Pbx homeoproteins during pancreatic development. Dev Biol.

[B61] Ohlsson H, Karlsson K, Edlund T (1993). IPF1, a homeodomain-containing transactivator of the insulin gene. Embo J.

[B62] Dillon N (2006). Gene regulation and large-scale chromatin organization in the nucleus. Chromosome Res.

[B63] Turque N, Plaza S, Radvanyi F, Carriere C, Saule S (1994). Pax-QNR/Pax-6, a paired box- and homeobox-containing gene expressed in neurons, is also expressed in pancreatic endocrine cells. Mol Endocrinol.

[B64] Kleinjan DA, Seawright A, Childs AJ, van Heyningen V (2004). Conserved elements in Pax6 intron 7 involved in (auto)regulation and alternative transcription. Dev Biol.

[B65] Vega http://vega.sanger.ac.uk/Danio_rerio/.

[B66] Imagenes http://www.imagenes-bio.de/.

[B67] Kwan KM, Fujimoto E, Grabher C, Mangum BD, Hardy ME, Campbell DS, Parant JM, Yost HJ, Kanki JP, Chien CB (2007). The Tol2kit: a multisite gateway-based construction kit for Tol2 transposon transgenesis constructs. Dev Dyn.

[B68] Fisher S, Grice EA, Vinton RM, Bessling SL, McCallion AS (2006). Conservation of RET regulatory function from human to zebrafish without sequence similarity. Science.

[B69] Fisher S, Grice EA, Vinton RM, Bessling SL, Urasaki A, Kawakami K, McCallion AS (2006). Evaluating the biological relevance of putative enhancers using Tol2 transposon-mediated transgenesis in zebrafish. Nat Protoc.

[B70] Hauptmann G, Gerster T (1994). Two-color whole-mount in situ hybridization to vertebrate and Drosophila embryos. Trends Genet.

[B71] Mavropoulos A, Devos N, Biemar F, Zecchin E, Argenton F, Edlund H, Motte P, Martial JA, Peers B (2005). sox4b is a key player of pancreatic alpha cell differentiation in zebrafish. Dev Biol.

[B72] Milewski WM, Duguay SJ, Chan SJ, Steiner DF (1998). Conservation of PDX-1 structure, function, and expression in zebrafish. Endocrinology.

[B73] Argenton F, Zecchin E, Bortolussi M (1999). Early appearance of pancreatic hormone-expressing cells in the zebrafish embryo. Mech Dev.

[B74] Korzh V, Sleptsova I, Liao J, He J, Gong Z (1998). Expression of zebrafish bHLH genes ngn1 and nrd defines distinct stages of neural differentiation. Dev Dyn.

[B75] Devos N, Deflorian G, Biemar F, Bortolussi M, Martial JA, Peers B, Argenton F (2002). Differential expression of two somatostatin genes during zebrafish embryonic development. Mech Dev.

[B76] Zhou X, Vize PD (2004). Proximo-distal specialization of epithelial transport processes within the Xenopus pronephric kidney tubules. Dev Biol.

[B77] Peers B, Leonard J, Sharma S, Teitelman G, Montminy MR (1994). Insulin expression in pancreatic islet cells relies on cooperative interactions between the helix loop helix factor E47 and the homeobox factor STF-1. Mol Endocrinol.

[B78] Schreiber E, Matthias P, Muller MM, Schaffner W (1989). Rapid detection of octamer binding proteins with 'mini-extracts', prepared from a small number of cells. Nucleic Acids Res.

[B79] Molina A, Di Martino E, Martial JA, Muller M (2001). Heat shock stimulation of a tilapia heat shock protein 70 promoter is mediated by a distal element. Biochem J.

