# Supplementary figures and images for: Expression of zebrafish pax6b in pancreas is regulated by two enhancers containing highly conserved cis-elements bound by PDX1, PBX and PREP factors
Source: BMC Dev Biol. 2008 May 16;8:53. doi: 10.1186/1471-213X-8-53 (PMC2409314; doi:10.1186/1471-213X-8-53)

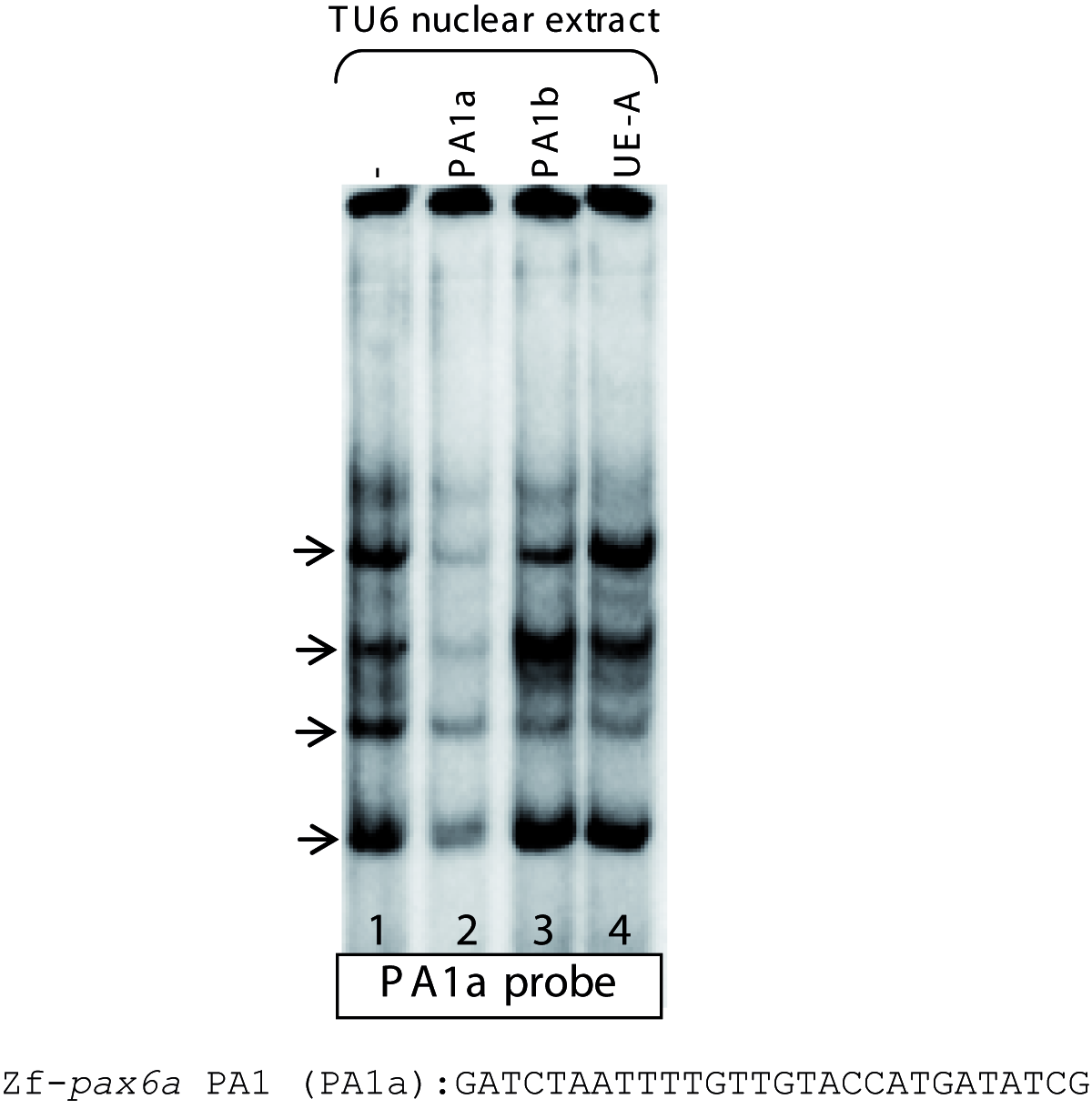

Supplement: Additional file 1 — No PBX-PREP-PDX1 complex is observed on homologous pax6a PA1 sequence. When we tested the homologous pax6a PA1 sequence (named PA1a), incubation with nuclear extract led to the formation of four major complexes (arrows, lane 1) which were displaced by adding a 100-fold molar excess of unlabeled PA1a (lane 2). Addition of unlabelled PA1b and somatostatin element UE-A, which bind PBX-PREP-PDX1 and PBX-PREP complex, respectively, do not displace the binding on the probe (lanes 3 and 4). This indicates that the proteins in the pancreatic extract which recognize the two cis-elements PA1b and UE-A do not recognize the homologous pax6a element. [file 1471-213X-8-53-S1.tiff]

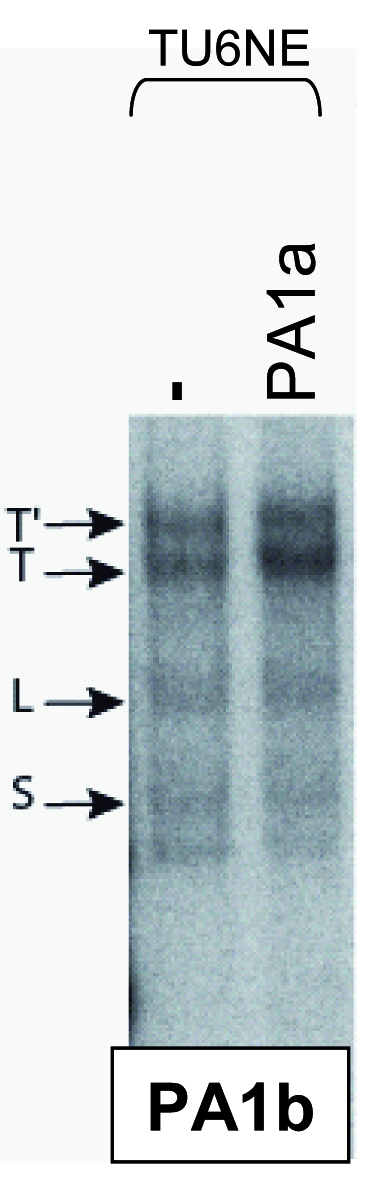

Supplement: Additional file 2 — Unlabeled PA1 of pax6a does not compete with PA1 of pax6b. To further demonstrate that PDX1 is unable to bind to PA1a, this sequence was used as competitor on PA1b probe. No competition was observed, suggesting that PDX1 binds specifically PA1 of pax6b and not PA1a. [file 1471-213X-8-53-S2.tiff]

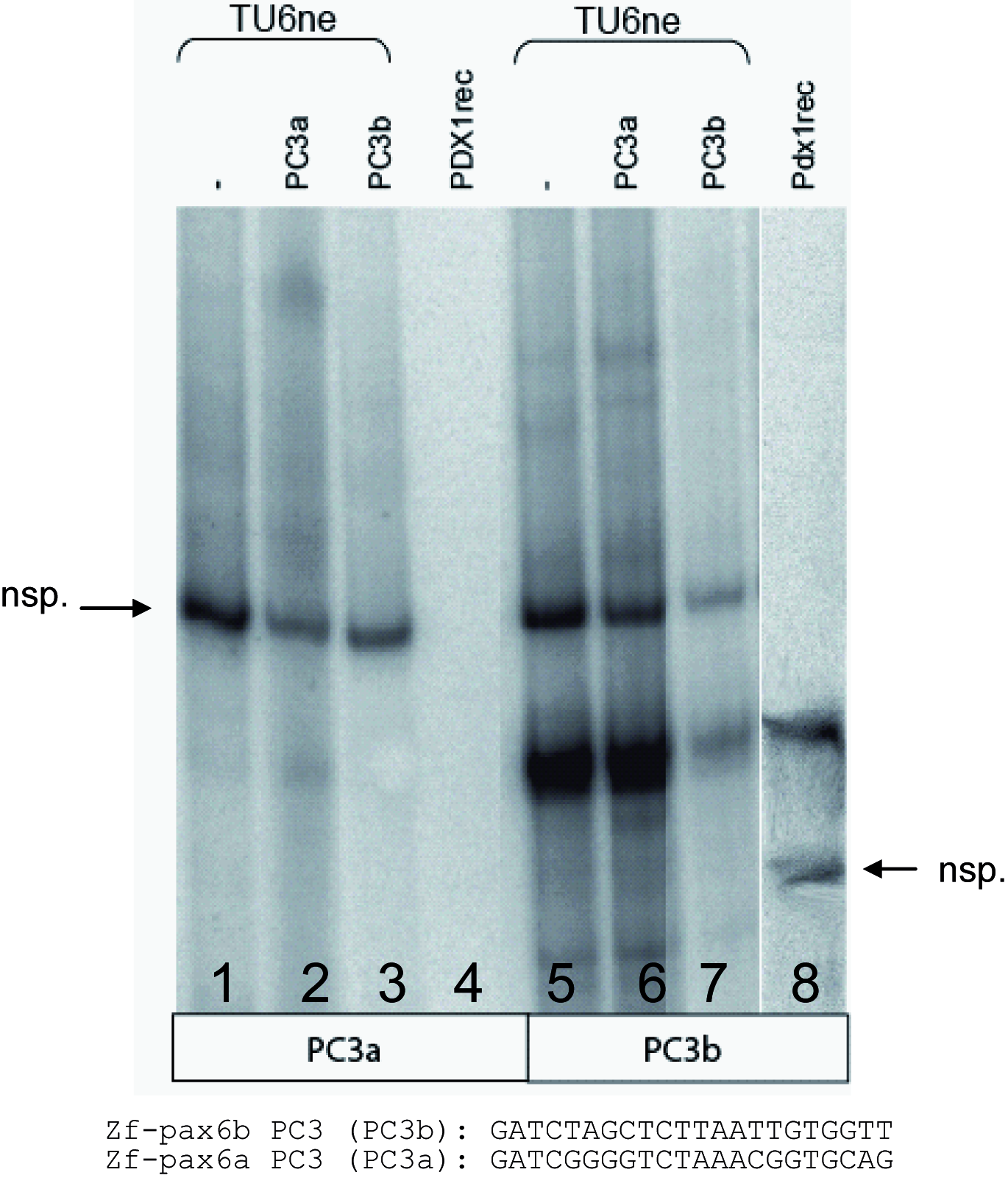

Supplement: Additional file 3 — PDX1 binds to PC3b and not to PC3a. EMSAs were performed on PC3 probes of pax6a (PC3a) and pax6b (PC3b) using nuclear extract of pancreatic TU6 cells. 10 ng of unlabeled oligonucleotides PC3a (lanes 2, 6), and PC3b (lanes 3, 7) were added as competitors. Abbreviations: PBX1a and PREP1, recombinant PBX1a and PREP1 proteins produced in vitro in a reticulocyte extract; PDX1 rec, PDX1 recombinant. [file 1471-213X-8-53-S3.tiff]
